# Simultaneous Enzymatic Cellulose Hydrolysis and Product Separation in a Radial-Flow Membrane Bioreactor

**DOI:** 10.3390/molecules27010288

**Published:** 2022-01-04

**Authors:** Saleha Al-Mardeai, Emad Elnajjar, Raed Hashaikeh, Boguslaw Kruczek, Bart Van der Bruggen, Sulaiman Al-Zuhair

**Affiliations:** 1 Chemical and Petroleum Engineering, UAE University, Al Ain 15551, United Arab Emirates; 201790743@uaeu.ac.ae; 2 Mechanical Engineering, UAE University, Al Ain 15551, United Arab Emirates; eelnajjar@uaeu.ac.ae; 3 Mechanical Engineering, New York University Abu Dhabi, Abu Dhabi 129188, United Arab Emirates; rh143@nyu.edu; 4 Chemical and Biological Engineering, University of Ottawa, Ottawa, ON K1N 6N5, Canada; bkruczek@uottawa.ca; 5 Department of Chemical Engineering, KU Leuven, 3000 Leuven, Belgium; bart.vanderbruggen@kuleuven.be

**Keywords:** membrane bioreactor, enzymatic hydrolysis, cellulose, product separation, product inhibition, alkaline pretreatment

## Abstract

Hydrolysis is the heart of the lignocellulose-to-bioethanol conversion process. Using enzymes to catalyze the hydrolysis represents a more environmentally friendly pathway compared to other techniques. However, for the process to be economically feasible, solving the product inhibition problem and enhancing enzyme reusability are essential. Prior research demonstrated that a flat-sheet membrane bioreactor (MBR), using an inverted dead-end filtration system, could achieve 86.7% glucose yield from purified cellulose in 6 h. In this study, the effectiveness of flat-sheet versus radial-flow MBR designs was assessed using real, complex lignocellulose biomass, namely date seeds (DSs). The tubular radial-flow MBR used here had more than a 10-fold higher membrane surface area than the flat-sheet MBR design. With simultaneous product separation using the flat-sheet inverted dead-end filtration MBR, a glucose yield of 10.8% from pretreated DSs was achieved within 8 h of reaction, which was three times higher than the yield without product separation, which was only 3.5% within the same time and under the same conditions. The superiority of the tubular radial-flow MBR to hydrolyze pretreated DSs was confirmed with a glucose yield of 60% within 8 h. The promising results obtained by the novel tubular MBR could pave the way for an economic lignocellulose-to-bioethanol process.

## 1. Introduction

Lignocellulose is the most abundant biomass, with an estimated yearly availability of 200 × 109 tons. Ideally, by using lignocellulose as feedstock, more than 400 billion liters of bioethanol can be produced annually [1]. The bioconversion process of lignocellulose to valuable chemicals, including bioethanol, has attracted increasing attention as a sustainable alternative source for producing liquid fuels. Currently, bioethanol production mainly depends on biomass that is readily used as a food stock. For example, South America uses sugar cane, corn is used in North America, whereas wheat and beet molasses are used in Europe. However, consuming food stock, also known as first-generation feedstock, for energy generation is argued to negatively affect food insecurity, which is a major global future challenge [1]. However, lignocellulosic waste, such as date seeds (DSs), does not compete with food sources, and using them is considered a waste management process. As far as the dates industry is concerned, all non-edible parts of the date palm tree impose an economic and environmental load on society, and converting them to energy is an area of interest [2].

Bioethanol production from lignocellulosic materials is a multiple-step process, in which hydrolysis is considered the heart of the process and the limiting step. Among the different methods proposed to hydrolyze the cellulose component in lignocellulose to fermentable glucose, enzymatic hydrolysis is favored due to its milder conditions, less energy consumption, and elimination of toxic waste. To have a cost-effective and economically feasible process, 4% *w*/*w* ethanol has to be produced before entering distillation, which requires around 8% *w*/*w* of produced glucose [3]. This necessitates a high solid loading of above 15% on a dry basis or an increase in the enzyme loading to enhance cellulose conversion and increase glucose production [4]. Continuous stirred reactors (CSTRs) in series were used for enzymatic hydrolysis of cellulose from creeping wild ryegrass pretreated with dilute sulfuric acid. The enhancement of the conversion and production yield by increasing the solid loading was evaluated. Continuous feeding with high loading using multiple CSTRs in series was found to enhance volumetric productivity; however, cellulose conversion was not improved [5]. The high solid loading, however, causes another complication during the reaction, which is an increase in the viscosity of the reaction medium that lowers the binding between enzymes and substrates and increases the production of inhibitors [6]. In addition, the use of a soluble enzyme in a continuous system would render the overall process unfeasible, owing to the high price of the enzymes that are inviably lost with the product. Enzymatic hydrolysis is considered a cost-limiting step that hinders large-scale production. The enzyme cost represents around 28% of the bioethanol selling price and 20% of the total production cost [3,7]. The selling price of cellulases was reported to be around 9500 USD/gal, which makes them precious to be added in large quantities to enhance the yield or lose them by product inhibition [8]. To this end, on-site enzyme production was considered in bioethanol production plants to reduce the cost by up to 70% [6]. In addition, the ability to recycle enzymes while maintaining high activity can lead to a 60% reduction in the total cost of bioethanol production [9], which clearly shows the significance of the work presented in this paper.

Immobilization of enzymes on a solid support was suggested to facilitate easy retaining and reusing of the enzymes inside continuous reactors [10]. For example, cellulase was immobilized on magnetic nanoparticles and used to catalyze the hydrolysis of cellulose in a batch reactor. The immobilized enzymes were successfully reused for multiple cycles, which is essential for a cost-effective process. However, glucose production decreased by over half and a quarter in the second and third cycles, respectively, as compared to the first cycle [11]. In another study, cellulases were also immobilized on iron oxide nanoparticles and used in a batch reactor for the hydrolysis of sugarcane bagasse pretreated with sulfuric acid. A similar drop in conversion was reported, in which the conversion decreased from 72% in the first cycle to 52% in the second. This shows that although immobilization can be considered a good approach to retaining the enzyme inside the reactor, it is not suitable to be used in conventional reactor systems for cellulose hydrolysis, since the enzyme would still be subjected to deactivation by the produced sugars, which results in a drop in stability [12]. In addition to using the enzyme in immobilized form, its activity drops compared to free enzymes [13]. This is mainly because immobilized enzymes are not suitable with heterogeneous substrates, such as cellulose, that necessitate the diffusion of the enzymes through the substrate matrix. This problem becomes more evident when the enzyme is immobilized on porous beads, which are easier to retain than nanoparticles.

Membrane bioreactors (MBRs) have been proposed as a better alternative to conventional continuous reactors to concurrently solve the product inhibition problem and allow enzyme reuse while used in free form. MBRs of different configurations have been tested for the enhancement of enzymatic hydrolysis of cellulose from different sources. The use of ultrafiltration membranes allowed a continuous selective permeation of the small product molecules while retaining the active enzymes inside the reaction for multiple cycles [14]. Separate reaction and separation units were tested to enhance enzymatic hydrolysis of pretreated microcrystalline cellulose at 50 °C. In this system, a stirred tank reactor containing the enzyme and the substrate was used and the effluent was subjected to semi-continuous product removal using a separate filtration unit containing a polyethersulfone (PES) membrane with a molecular cut-off (MWCO) of 10 kDa. Using this configuration, 95% conversion was achieved within 4 h [15]. Despite the positive results, the pumping of the enzyme between the reaction and separation units negatively affects the enzyme stability. Therefore, and to avoid using two separate units and to eliminate the internal pumping, a new configuration was proposed in which the filtration membrane was installed inside the reactor system, at the bottom of the reaction cell, known as the dead-end filtration system. Besides eliminating the internal pumping, the in situ continuous product separation had another positive effect on pushing the reaction forward. However, the system suffered from insufficient mixing due to substrate accumulation at the bottom, near the membrane surface, which also caused severe membrane fouling [16,17,18]. In our previous work [19], an MBR with an inverted dead-end concept was tested using standard cellulose as a substrate. Enhanced enzymatic hydrolysis was achieved by continuous removal of the product with insignificant membrane fouling or damage. Under the same reaction conditions, the cellulose conversion in the MBR increased by a factor of 6.5 compared to the case of no product separation. At the optimum conditions of 2.67 g/L of substrate concentration and 0.8 mL/min water flux using a PES with an MWCO of 10 kDa, a product yield of 86.7% was achieved in 6 h of reaction. By placing the membrane above the reaction cell, substrate deposition and protein fouling were minimized and no changes in the membrane structure were detected after prolonged usage of the system. In this study, we aim at validating the applicability of the newly designed and constructed inverted dead-end MBR using real waste lignocellulosic biomass, namely DSs, which are more complex.

In another configuration, a polyethylene hairy membrane, of an unidentified membrane cut-off, was used to absorb both substrate and enzymes. The hairy membrane was installed on a stainless-steel tube with a pore size of 0.1 µm and constructed in a tubular design. The substrate and enzymes were recirculated using a peristaltic pump in the annulus of a tubular reactor, while the membrane surrounded the inner tube to which the produced glucose diffused and was collected by another peristaltic pump from a permeate outlet. Pressure was adjusted through an air inlet in the annulus side of the reactor. The membrane was classified as ultrafiltration, due to its ability to separate the substrate and enzyme molecules. The enzymatic hydrolysis was assumed to be enhanced due to product removal and reverse adsorption of the substrate and enzymes [20]. This design, however, has several drawbacks, most importantly being prone to sever fouling. In addition, having the substrate in the annulus side limits its loading concentration and efficient mixing. Results showed a significant difference in flux as a function of transmembrane pressure (TMP) when pure permeating water was used as compared to the case of the water contained cellulose powder. For example, at a pressure of 100 kPa, water flux was measured to be 30 L/m^2^ bar h, whereas with 2.5% (*w*/*v*) Solka Folc cellulose solution, the flux reduced to 5 L/m^2^ bar h. The flux was also found to be proportional to the TMP only at low pressures (in ranges less than 200 kPa). This can be only attributed to fouling caused by the solute adsorption on the hairy membrane. Furthermore, the membrane was not efficient in rejecting the cellulase, and 6% of the initial enzyme activity was reported in the permeate after 10 h [20], which defeats the original purpose of using a membrane to retain the enzyme and maintain it active inside the reactor, thus reducing the cost and facilitating large-scale production. In addition, the continuous recirculating pumping of the substrate slurry makes the process difficult to scale up, and pumping the enzymes accelerates their deactivation.

The propose of this study therefore was to propose, design, and test a novel tubular, radial MBR design that can enhance cellulose conversion, while eliminating the drawbacks of all previous design. A PES ultrafiltration membrane, with a 10 kDa cut-off, was installed in the novel radial MBR, which created an inner cylinder, where the reaction would take place. This arrangement provided a much higher diffusion area per reaction volume as compared to the dead-end filtration system. The larger diffusion area is expected to enhance the product separation and further enhance the enzymatic hydrolysis. The membrane used was PES ultrafiltration, which was proven in our previous study to permeate glucose and totally reject enzymes, thus avoiding losing expensive enzymes [19]. In addition, slurry and enzyme pumping was avoided, while providing efficient mixing, which plays a crucial role in enhancing the enzyme–substrate binding and reducing fouling. The presented novel radial MBR has the potential to be scaled up for larger production. The effectiveness of using the tubular MBR was evaluated using DSs as a substrate. The positive results of this work could pave the way for economic bioethanol production from lignocellulosic waste.

## 2. Results

### 2.1. Biomass Characterization and the Effect of Pretreatment on the Substrate

The fresh DSs, after removing the extractives, were characterized. Cellulose, hemicellulose, and lignin contents were determined to be 47 ± 0.7%, 28 ± 0.4%, and 25 ± 0.6%, respectively. This analysis falls within the typical ranges of the constituents’ most lignocellulosic materials [21]. To confirm the lignin content and to investigate the efficiency of lignin removal pretreatment, Klason lignin and acid-soluble lignin were quantified. Klason lignin and acid-soluble lignin in the fresh untreated DSs were determined to be 24.01% ± 2.47 and 0.16% ± 5.59 × 10^−5^, respectively. The determined total lignin of 24.17% was in agreement with that found by the constituents’ analysis. Klason lignin and acid-soluble lignin in the lignin-removed samples were found to be 16.6% ± 2.6 and 3.95 × 10^−4^% ± 1.8 × 10^−4^, respectively. Hence, over 30% of total lignin was removed, which confirms the effectiveness of the alkaline pretreatment. The method has been reported as an efficient approach for lignin removal and biomass swelling to enhance the enzymatic hydrolysis of lignocellulosic material, while preventing the solvation of hemicellulose [22]. The biomass type, alkaline concentration, temperature, and pretreatment time are factors that affect the percentage removal of lignin. By using alkaline treatment at 4% NaOH for 1 h at room temperature, 15.68% of lignin content was removed from DSs [23]. Operating at a higher temperature of 80 °C and 2% NaOH, a higher lignin removal of 70% was achieved from herbaceous lignocellulose, such as wheat straw, corn straw, and sugar bagasse. However, the increase in temperature was less effective with hardwood and softwood biomass, achieving a removal of 39.6% and 16%, respectively [24].

**Figure 1:**
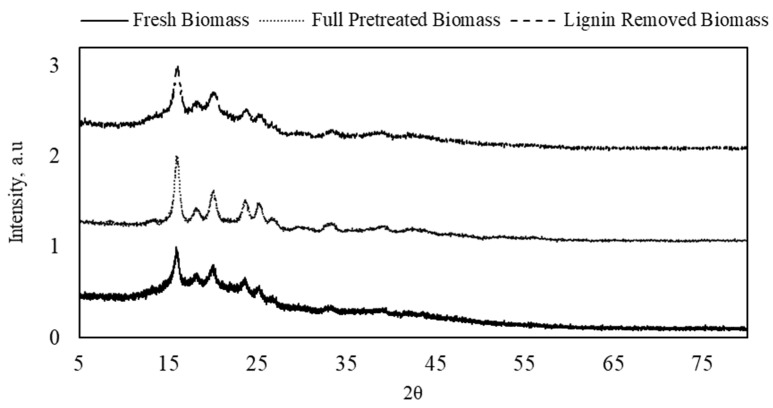
X-ray diffraction spectra of fresh, full-pretreated, and lignin-removed DSs.

The effects of the full pretreatment and lignin removal treatment on the crystallinity of the DSs were examined with XRD, as shown in Figure 1. The analysis showed a slight increase in the intensity after both treatments, which indicates an increase in the crystallinity. The peaks at 16° and 23° reflect crystalline cellulose type I, while cellulose type II is presented at 20°. The intensity of those peaks increased in both full-pretreatment and lignin-removed DSs biomass, which indicates a higher crystallinity degree in the structure of the treated fibers due to the removal of lignin [25]. A similar increase in the intensity of peaks that represent crystalline cellulose was also observed with DSs from *Phoenix dactylifera* L. after lignin and hemicellulose removal [26]. It was also noticed that the increase in peak intensity with full pretreatment (i.e., removing both lignin and hemicellulose) was higher than that with lignin removal only. This suggests that the acid used in the full pretreatment may not have hydrolyzed hemicellulose only but also the amorphous parts of the cellulose structure, resulting in increased crystallinity [27]. As a result, the peaks at 25° and 26°, which reflect cellulose crystallinity and crystalline carbon, respectively, appeared only in the fully pretreated sample.

**Figure 2:**
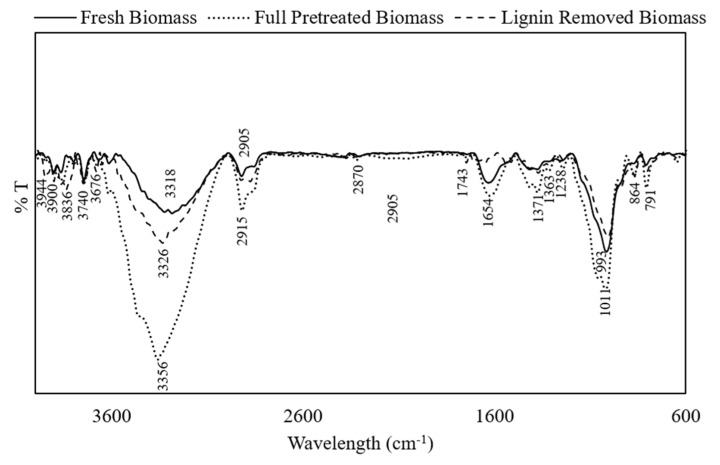
Fourier-transform infrared spectra of fresh, full-pretreated, and lignin-removed DSs.

FTIR analysis was carried out to investigate the effect of full and lignin-removing pretreatments on the composition and functional groups in DSs. Figure 2 shows the FTIR spectra of fresh DSs, full-pretreated DSs, and lignin-removed DSs. The peaks between 4000 and 2995 cm^−1^ are assigned to hydrogen-bonded OH stretching vibrations [28]. Peaks at 2905 and 2855 cm^−1^ are assigned to asymmetric and symmetric C–H stretching in methyl and methylene groups, respectively, and the peak at 2870 cm^−1^ represents aliphatic CH stretching [29], to which all three main constituents, cellulose, hemicellulose, and lignin, contribute. The peak at 1654 cm^−1^ reflects the C=C or C=N vibration in the aromatic region, which is influenced by the lignin [28]. The band at 993 cm^−1^ represents the C–O–C glycosidic bond vibration, and C–C stretching is assigned to the peak at 791 cm^−1^, which are related to the cellulose component [30]. After partial pretreatment, where lignin is removed, a similar spectrum to that of the fresh DSs was observed but with the appearance of two peaks at 1743 and 864 cm^−1^. The former is assigned to the C=O bond that can be due to either the acetyl or the ester group found in hemicellulose, while the latter represents the C–H rocking vibration of cellulose [31,32,33]. The appearance of those two peaks are signs of structural distribution and that lignin removal allowed the exposure of both cellulose and hemicellulose. By comparing the spectra of the full-pretreated DSs, it was observed that peaks at 1371 and 1338 cm^−1^ appeared, which are assigned to CH stretching of cellulose, which indicates the biomass structural deformation after hemicellulose and lignin removal. The appearance of the band at 1238 cm^−1^ can represent either C–O–H deformation or C–O phenolic stretching that might reflect traces of lignin found in the biomass. The appearance of a band at 864 cm^−1^ represents, as mentioned earlier, the C–H rocking vibration of cellulose [31].

**Figure 3:**
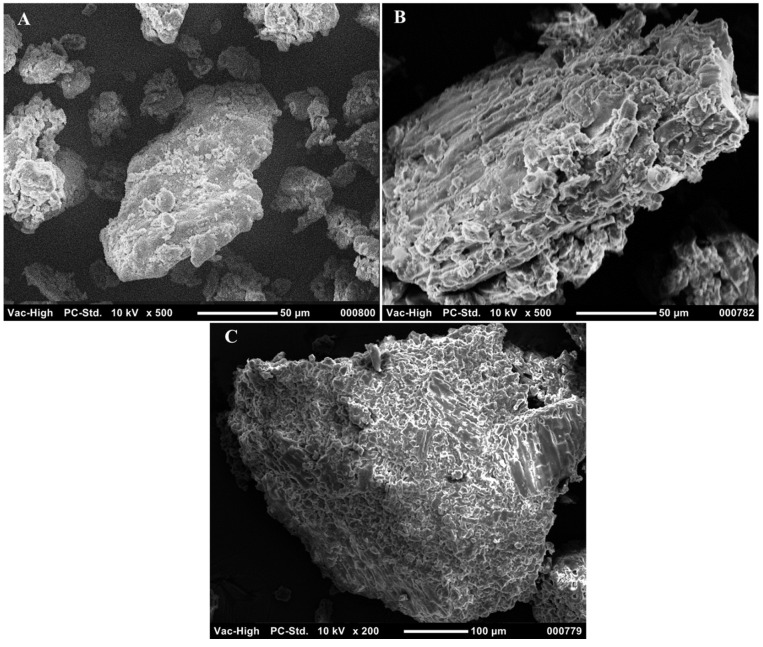
Scanning electron microscopy images of (**A**) fresh, (**B**) full-pretreated, and (**C**) lignin-removed DSs.

SEM was used to analyze the morphological changes as a result of full pretreatment and lignin removal pretreatment, as shown in Figure 3. The fresh DS biomass, shown in Figure 3A, was characterized by a smooth and solid surface. The effect of full pretreatment, including acid and NaOH treatment, is shown in Figure 3B. It can be seen that the structure was disrupted, due to the action of NaOH, leaving holes that can be observed on the surface of the molecules, while elongated, structural cracks indicate the severe action of acid treatment, attributed to the partial hydrolysis of the amorphous part of the biomass. Images of the lignin-removed DS biomass, treated with only NaOH, are shown in Figure 3C. It can be seen that the structure contained holes that indicate the disruption of the matrix due to lignin removal. However, unlike with the full-treated samples, with lignin removal only, the samples maintained a similar overall structure without cracks [28].

### 2.2. Enzymatic Hydrolysis with Product Separation

Acid-based pretreatment was used to remove both hemicellulose and lignin to disrupt the lignocellulose structure and enhance the enzyme accessibility to cellulose. A preliminary test was performed to evaluate the need for the hemicellulose-removing step. The glucose yield from enzymatic hydrolysis of DSs pretreated by the removal of lignin and hemicellulose was found to be close to that obtained using DSs treated with lignin removal only. With complete pretreatment, the glucose yield after 8 h of hydrolysis was 3.2%. Under the same conditions, the yield obtained from DSs with lignin removal only was 3.5%. Similar results were also observed when comparing the hydrolysis yield of corn stalk biomass fully pretreated by lignin and hemicellulose removal and partially pretreated by lignin removal only. After 96 h of hydrolysis, the cellulose conversion in the fully pretreated samples was 70.3%, whereas it reached 100% in partially pretreated samples. Delignification only of loblolly pine biomass was also shown to increase the cellulose hydrolysis conversion after 72 h by 88% as compared to untreated samples, where cellulose conversion was only 16%. [34]. The lower conversion of the fully pretreated samples is attributed to the use of acid, which, in addition to removing hemicellulose, dissolves some of the amorphous portion of cellulose. This increases the percentage of the crystalline cellulose in the fully pretreated sample observed in the XRD analysis, which results in a drop in the conversion yield [28]. Therefore, all subsequent experiments using the MBR were carried out using DS samples partially pretreated with lignin removal only.

**Figure 4:**
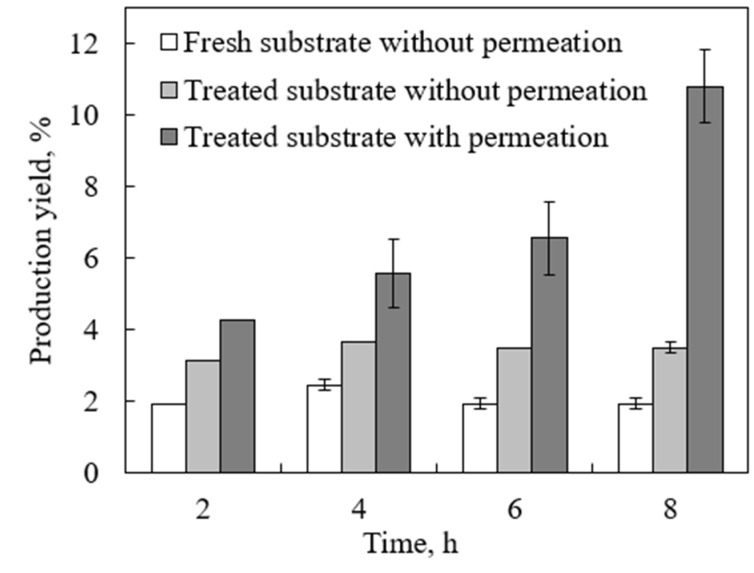
Glucose production yield from fresh and lignin-removed DSs with and without permeation in an inverted dead-end MBR using substrate and enzyme concentrations of 13.3 g/L and 252 FPU/mL, respectively, at pH 5, 48 °C, and a water flux of 0.8 mL/min.

In our previous study, the enzymatic hydrolysis of standard cellulose with simultaneous product removal in an inverted dead-end MBR was tested and modeled [19]. The results clearly showed a significant enhancement in the reaction rate and production yield with the continuous in situ removal of the products. In this study, the developed inverted dead-end MBR was tested on real lignocellulosic biomass, namely DSs, which are more complex and harder to degrade than standard cellulose. Initially, the glucose production yield by enzymatic hydrolysis of fresh DSs was compared to lignin-removed DSs without product separation. As shown in Figure 4, by using lignin-removed DSs, the yield after 8 h of reaction reached 3.2% compared to less than 2.1% with fresh DSs. This further confirms the significance of lignin in enhancing the cellulase accessibility to cellulose that enhances the hydrolysis.

However, without product separation, the production yield tended to reach a plateau after 4 h and no increase was observed. This is mainly due to the glucose inhibition effect, which was also observed with standard cellulose used in our previous work [19]. With continuous product separation, however, the yield significantly increased to 10.8%. This further proves the significance effect of product removal on enhancing the reaction rate and yield, similar to standard cellulose. The production yield using the real lignocellulosic biomass was 10.8% compared to 29.9% with standard cellulose under the same conditions. The higher yield in the latter is attributed to the fact that it is composed of pure hydrolysable cellulose, which is easily accessible to cellulase.

These findings agree with previously reported results of cellulose hydrolysis enhancement by simultaneous product separation. It was reported that an increase in conversion by up to 40% could be achieved using a dead-end MBR system as compared to the case without product separation in a conventional batch reactor [35]. For example, using a dead-end MBR with a flat-sheet polysulfone membrane, 10 kDa, the enzymatic hydrolysis conversion of α-cellulose after 48 h increased to 53%, compared to 35% in a batch reaction under same operating conditions [36]. However, this MBR configuration suffered from membrane fouling, necessitating a high transmembrane pressure to increase permeation, which was avoided using the inverted dead-end MBR, as explained in our previous paper [19]. A dead-end filtration module, with a polyethersulfone membrane of a smaller MWCO of 2 kDa, was used for semi-continuous product removal, and enzymatic hydrolysis of cotton cellulose increased to 27% as compared to only 5% in a batch system after 72 h [37]. In this system, however, intermittent product removal was carried out rather than continuous removal. The lower conversion in this study could also be attributed to the different type of cellulases used.

**Figure 5:**
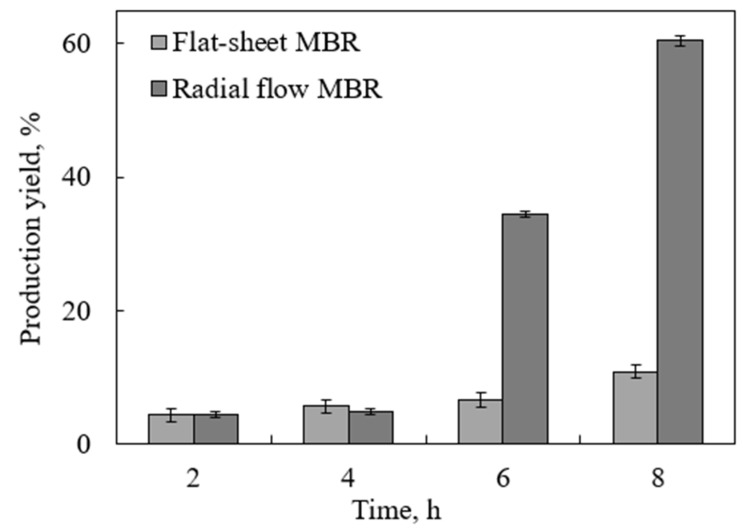
Glucose production yield from lignin-removed DSs using an inverted dead-end MBR and a radial-flow MBR using substrate and enzyme concentrations of 13.3 g/L and 252 FPU/mL, respectively, at pH 5, 48 °C, and a water flux of 0.8 mL/min.

The most important finding of this work is testing the novel tubular MBR with a radial-flow MBR. The new design provided a larger diffusional surface area, which increased the glucose diffusion and in turn resulted in further enhancement of the reaction rate and production yield. This idea was first suggested by a co-author of this paper [38] for a simulated case. In this work, however, the reactor was physically built and challenged by a real test. The glucose production yield from the enzymatic hydrolysis of partially treated DSs is shown in Figure 5, as compared to that achieved using the inverted dead-end MBR, under the same conditions. The superiority of the new design was overwhelming, with the yield reaching up to 60% within 8 h. In the first 4 h, the performance of both reactors was almost identical. This is because the concentration of the produced glucose was not high enough to cause inhibition. However after 6 h, the effect of glucose removal became more significant, and the superiority of the radial MBR over the inverted dead-end MBR became more evident. The enhancement in the yield in the radial MBR is attributed to the increase in the diffusion area. The tubular membrane surrounding the reaction area throughout the reactor facilitated more product separation and pushed the reaction in the forward direction compared to the limited diffusion area provided in the flat-sheet MBR. In addition, at the substrate concentration used, no mixing problems were encountered, which suggests that the reactor can handle higher concentrations, a major challenge facing our previous inverted dead-end design.

The conversion achieved using the novel tubular bioreactor reported in this work was close to that reported using the polyethylene hairy membrane tubular reactor used for the hydrolysis of Mavicell cellulose, which was 70% in 10 h. However, the substrate used, Mavicell cellulose, was pure cellulose, which was further thermally treated at 120 °C for 20 min to disrupt its structure. In this work, a similar high conversion was achieved using a more complex substrate, DSs, which was pretreated by lignin removal only, a much milder condition, leaving the cellulose intact. Indeed, when untreated Mavicell cellulose was used, the conversion in the hairy membrane tubular reactor dropped significantly to 20% under the same operating conditions [20]. The membrane fouling caused by substrate and enzyme deposition, and the enzyme loss and deactivation through continuous pumping, further contributed to lower conversion. The results clearly show the superiority of the radial MBR proposed in this work for enhancing enzymatic hydrolysis of real complex substrates, such as lignocellulose. The positive results from this work are promising, and the work will continue to model and optimize the process.

## 3. Materials and Methods

### 3.1. Chemicals and Enzymes

Cellulase from *Trichoderma reesei* was purchased from Sigma-Aldrich, St. Louis, MO, USA. The enzyme activity was measured to be 1800 units/g. The activity unit is defined as the concentration of glucose, in g, produced within 1 h from the filter paper at 37 °C and a pH of 5. Dinitrosalicylic acid (DNS), glucose (99.5% purity), Bradford reagent for protein detection, and all other chemicals were purchased from Merck, USA. Sodium acetate (99%) and acetic acid (99%) were used to prepare a 0.1 M acetate buffer (pH 5) for controlling the pH of the system. Hydrochloric acid (HCl), 37%, and sodium hydroxide (NaOH) were purchased from Sigma-Aldrich (St. Louis, MO, USA). Microdyn Nadir hydrophilic polyethersulfone (PES) ultrafiltration membranes (297 × 210 mm) with a 10 kDa MWCO and a thickness of 230 µm were obtained from Sterlitch, Auburn, WA, USA.

### 3.2. Biomass Preparation and Analysis

DSs from Allig dates were obtained from Liwa Company in the Abu Dhabi, United Arab Emirates. The DSs were washed with distilled water and then sun-dried and ground. The dried, ground DSs were screened, and those with the size of 180 µm were used in this work. Extractives are components that naturally exist in, or are attached to, biomass [39]. To avoid interference of the free extractives in the pretreatment and enzymatic hydrolysis processes and for an unbiased evaluation of the developed MBR, free extractives were removed in a Soxhlet extraction unit using ethanol for 8 h. To characterize the substrate, the acid–base isolation method, described by [40], was used to quantify the three main constituents, namely cellulose, hemicellulose, and lignin. Briefly, 10 g of substrate was mixed with 200 mL of 0.1 M HCl while being heated at 100 °C for 2 h with stirring at 150 rpm. The mixture was vacuum-filtered to separate residues containing cellulose and lignin from the liquid containing hemicellulose. The residues were then washed with 20 mL of distilled water to remove any remaining hemicellulose left, followed by air-drying overnight. The dried residues were then treated with 200 mL of 0.1 M NaOH for 2 h at 100 °C under stirring at 150 rpm. Subsequently, the mixture was vacuum-filtered to separate residues containing cellulose from the lignin that was solubilized in NaOH. The residues were further washed with 20 mL of 0.1 M of NaOH and air-dried.

The hydrolysis of fully treated samples, in which both lignin and hemicellulose were removed, was compared to that of partially treated samples, in which only lignin was removed while keeping the hemicellulose. For the latter case, 10 g of the substrate sample was treated only with 200 mL of 0.1 M NaOH for 2 h. As shown in Section 3.2, the removal of hemicellulose did not result in any enhancement in hydrolysis, and a similar yield was obtained to that obtained using fresh DS biomass. Therefore, all the substrates used in all subsequent experiments were on partially treated (lignin-removed) DSs.

### 3.3. Enzymatic Hydrolysis with Product Separation

In this study, enzymatic hydrolysis of DSs at a concentration of 13.3 g/L and a water flow of 0.8 mL/min was investigated using 252 FPU/mL of enzyme concentration at 48 °C and an agitation speed of 450 rpm. These conditions were the optimum conditions determined in our previous work using standard cellulose [19]. All experiments were conducted in triplicate, and the results presented in all figures are the average values, with the standard deviations shown as error bars.

### 3.4. MBR Design

#### 3.4.1. Inverted Dead-End Filtration MBR

The inverted dead-end filtration MBR presented in our previous study [19] was used in this work to validate its applicability on real lignocellulosic waste biomass. The MBR consists of two zones separated by a PES membrane. The reaction took place in the lower zone, whereas the upper zone contained only distilled water with an adjusted pH of 5. The temperature inside the reaction zone was controlled using insulated heating tape that surrounded the bottom zone, fitted with a thermocouple connected to a temperature controller. To avoid temperature fluctuations, the bottom zone was further covered with wool insulation and wrapped with aluminum foil. Water at the same pH and temperature of the reaction zone was introduced into the bottom zone, diffused through the membrane to the upper zone, and collected in a beaker. The water flow was maintained within a range that was found not to cause physical damage to the membrane. More details about the design and operation of the inverted dead-end MBR can be found in our previous work [19]. The system was tested using DSs at a concentration of 13.3 g/L and a water flow of 0.8 mL/min, which were the optimum conditions determined in our previous work using standard cellulose. All experiments were conducted in triplicate, and the results presented in all figures are the average values, with the standard deviations shown as error bars.

#### 3.4.2. Radial-Flow MBR

**Figure 6:**
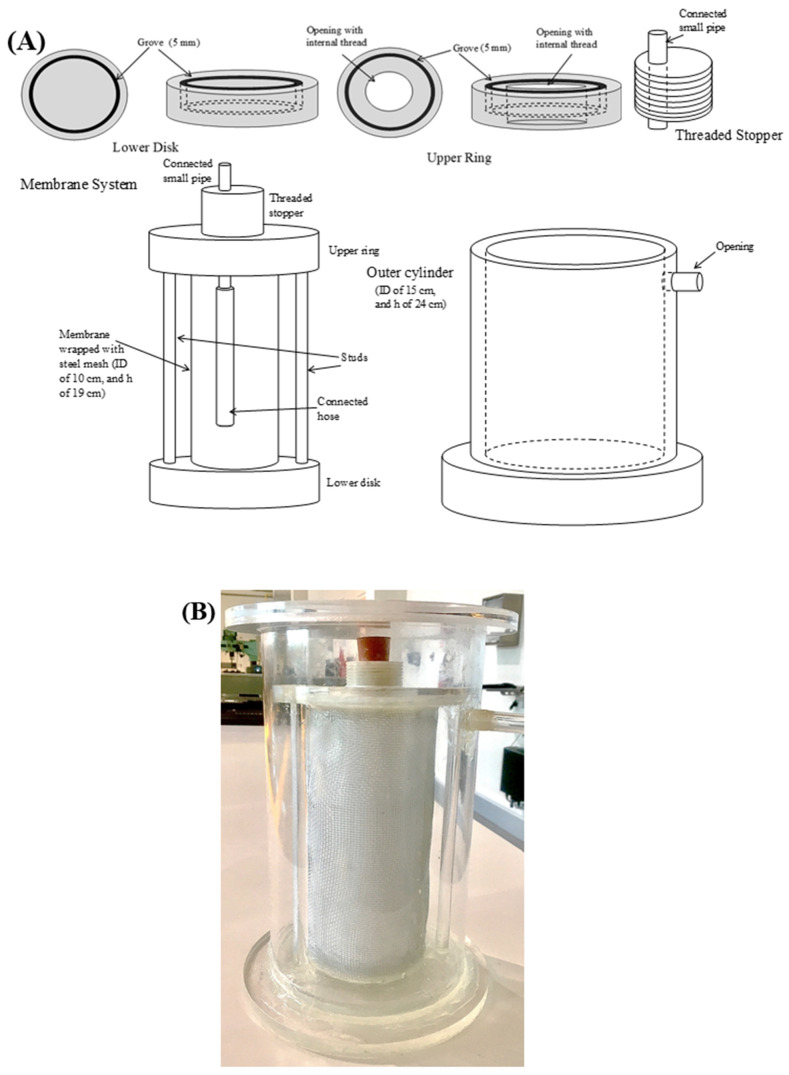
(**A**) Schematic diagram of the radial-flow tubular MBR and (**B**) photo of the assembled tubular MBR.

The radial-flow MBR was designed with an inner zone jacketed with outer cylinder, as shown in Figure 6. The wall of the inner zone, of an internal diameter, ID, of 10 cm and a height, h, of 19 cm, surrounded by an outer cylinder of ID 15 cm and h 24 cm. The reaction was carried out in the inner zone, where enzymes and substrate were charged, while the outer cylinder contained only distilled water with a pH similar to that in the reaction zone. The walls of the inner reaction zone were composed of the PES membrane trapped between two woven stainless-steel wire meshes to provide physical support to was attached to the upper and lower disks by tight epoxy adhesive glue and further sealed with silicone to eliminate any possibility of leakage. This was confirmed by subjecting the system to a leakage test before being used in the experiment. The reaction zone was agitated using a magnetic stirrer at 450 rpm, which was shown in our previous work on an inverted dead-end filtration membrane to create enough turbulence for good mixing, while avoiding shear stress that could deactivate the enzymes [19]. The temperature of the reaction was maintained at 48 °C by placing the reactor on a hot plate, set at 48 °C and covering the outer cylinder with a heating tape (Thermolyne, Sigma) fitted with a thermocouple that was connected to a temperature controller (TC4S-14R). The reactor was further covered with wool insulation and wrapped with aluminum foil to minimize any heat loss and temperature fluctuation. Distilled water at the reaction temperature and adjusted pH 5 was introduced into the inner reaction zone using a peristaltic pump (Shenzhen, China), and it diffused through the membrane, with the produced glucose, to the outer zone. The water passed from the inner cylinder to the outer cylinder through the membrane in a radial direction and was collected in a beaker. The system was tested at the same conditions used for the inverted dead-end MBR. All experiments were conducted in triplicate, and the results presented in all figures are the average values, with the standard deviations shown as error bars.

**Figure 7:**
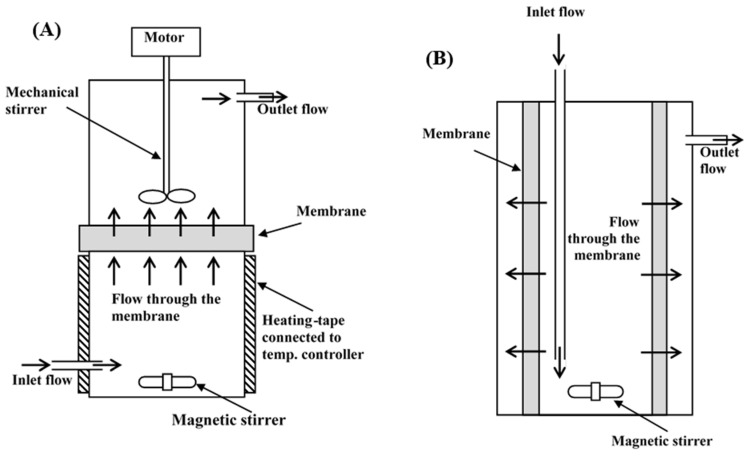
Flow diagram of (**A**) inverted dead-end and (**B**) radial-flow MBRs.

Flow diagrams of the inverted dead-end and radial-flow MBRs are shown in Figure 7. In the inverted dead-end MBR, glucose and water flowed from bottom to top across the membrane against gravity, as shown in Figure 7a. The active membrane surface area and specific area per volume of reaction in the inverted dead-end MBR were 44 cm^2^ and 0.06 cm^2^/cm^3^, respectively, whereas in the radial-flow tubular MBR, the flow took place throughout the entire membrane surface area surrounding the reactor, as shown in Figure 7b. The active membrane surface area and specific area per volume of reaction in the radial-flow tubular MBR were 578 cm^2^ and 0.62 cm^2^/cm^3^. It was clear that the radial flow allowed for more product separation, which shifted the reaction more toward the forward direction.

To start up the process in the radial-flow tubular MBR, the outer cylinder was first filled with a pH 5 buffer solution in distilled water at the desired temperature to reach a volume that was previously determined to cover the entire membrane area. A substrate solution in a pH 5 buffer at the same desired temperature was slowly added to the inner cylinder through the unglued threaded stopper while inserting the cylinder in the outer one. This step was carefully carried out to keep an equal volume level in both cylinders to avoid creating a pressure difference across the membrane. A buffer solution at the desired temperature then added through the inlet line to fill the inner cylinder completely, and then the stopper was threaded back and sealed with silicone to avoid leakage. Once a steady state was reached, the enzyme was added through the inlet line to start the reaction.

### 3.5. Analysis

#### 3.5.1. Glucose Analysis

The glucose concentration was measured using the DNS method, which measures the total amount of reducing sugars in a sample [41]. In this method, 45 µL of the sample was mixed with 40 µL of the DNS reagent and diluted with 315 µL of distilled water to reach a total volume of 400 µL. Then, the mixture was incubated at 100 °C for 5 min. To stop the reaction with DNS, the sample was incubated in ice for 10 min. The glucose was then quantified using a UV spectrophotometer (BMG SPECTROstar, Ortenberg, Germany) at 540 nm. The glucose concentration in the sample was determined by comparing the measured optical density against a calibration curve constructed using serial dilutions of a glucose solution of known concentration.

#### 3.5.2. Protein Analysis

Protein measurement was done using the Bradford reagent, which is an acidic Coomassie blue dye that binds stably to proteins. The protein concentration was quantified by measuring the optical density at 595 nm and compared to a calibration curve constructed using serial dilutions of a cellulase solution of known concentration.

#### 3.5.3. Lignin Analysis

The lignin content of fresh and lignin-removed DS samples was determined in accordance with the NREL LAP-003 protocol [42]. In this protocol, both acid-insoluble lignin, known as Klason lignin, and acid-soluble lignin were quantified. Briefly, 0.3 g of the extractive-free sample was treated with 72% sulfuric acid (H_2_SO_4_) at 30 °C for 2 h with mixing every 15 min. The solution was then diluted with 84 mL of distilled water to reach a 4% acid concentration, after which it was autoclaved for 1 h at 121 °C. The sample was then allowed to cool down before being vacuum-filtered to separate the liquid from the residues. The residual part, which contained the Klason lignin, was placed in a muffle furnace at 575 °C for 3 h to determine the ash content. The acid-soluble lignin was determined by measuring the absorbance of the liquid filtrate at 205 nm.

#### 3.5.4. Substrate Characterization

The changes in the structural morphology due to the removal of lignin were detected using scanning electron microscopy (SEM; JCM-5000; NeoScope, Peabody, MA, USA). Fresh, full-pretreated, and lignin-removed DS biomasses were coated with gold using a JFC-1600Auto Fine Coater (JEOL) to increase the conductivity of the non-conductive catalyst and to prevent the build-up of electrostatic charge at the specimen surface. The samples were inserted into the scanning electron microscope for observation.

To analyze the functional groups in fresh, full-pretreated, and lignin-removed DS biomasses, Fourier-transform infrared (FTIR) spectroscopy (IRTracer-100 FTIR spectrophotometer; Shimadzu, Kyoto, Japan) was used. The FTIR spectra were recorded on an attenuated total reflection Fourier-transform infrared (ATR-FTIR) spectrograph using a range of 600–4000 cm^–1^ with an average of 34 scans and a spectral resolution of 4 cm^–1^.

X-ray diffraction (XRD) analysis was also used to measure changes in the crystallography of the fresh, full-pretreated, and lignin-removed DS biomasses. XRD scans were performed with a 2θ range of 4–90°, a step size of 0.02°/s, a voltage of 40 kV, an intensity of 20 A, and Cu Kα radiation of 1.5406 Å.

## 4. Conclusions

In this study, the applicability of an inverted dead-end MBR, previously tested on standard cellulose, was evaluated using real lignocellulosic waste biomass, namely date seeds (DSs). It was shown that removing only lignin, while retaining hemicellulose, was sufficient pretreatment to expose the cellulose to the enzyme. The effect of the pretreatment on opening of the lignocellulose structure and exposing of the cellulose was confirmed using SEM, FTIR, and XRD analyses. Using an inverted dead-end MBR, continuous and simultaneous product separation was achieved during the reaction, which resulted in an enhanced glucose production yield. The enzymatic hydrolysis was further enhanced using a newly designed radial-flow MBR that provided a larger diffusion area. The results of this study represent a significant addition to MBR research for enhanced enzymatic hydrolysis of lignocellulose for bioethanol. In addition, it opens the door for further investigations of the effect of different operating conditions and system modeling and optimization. In addition, other types of membranes, such as ceramic membranes, that can provide better mechanical support can be investigated.

## Data Availability

The data presented in this study are available in the article.

## Sample Availability

Samples of the compounds are available from the authors.
